# Numerical modeling of fluid exchange between a collecting lymphatic vessel and the surrounding tissue

**DOI:** 10.1007/s00285-026-02431-0

**Published:** 2026-07-07

**Authors:** Marina Furkes, Sanjoy Saha, N. Keilany Lightsey, Donny Hanjaya-Putra, Martina Bukač

**Affiliations:** 1 Department of Applied Mathematics, Faculty of Electrical Engineering and Computing, University of Zagreb, Zagreb, 10000 Croatia; 2 Aerospace and Mechanical Engineering, University of Notre Dame, ND, 46556 IN USA; 3 Applied and Computational Mathematics and Statistics, University of Notre Dame, ND, 46556 IN USA

**Keywords:** Lymphatic system, Fluid-poroelastic structure interaction, Mathematical modeling, Oscillatory pumping mechanism

## Abstract

Lymphatic vessel dysfunction is increasingly recognized in metabolic diseases, contributing to edema, dyslipidemia, and tissue lipid accumulation. Type 2 diabetes has been experimentally linked to lymphatic vascular defects characterized by enhanced permeability resulting from low nitric oxide (NO) bioavailability. To examine the effects of this dysfunction, we developed a mathematical model of fluid exchange between a collecting lymphatic vessel and the surrounding tissue. The lymph flow is modeled using the Stokes equations for an incompressible, viscous fluid, while the collecting vessel is represented by a thin Koiter shell model. The main novelty of our approach is that the interstitium is described as poroelastic, and modeled using the Biot equations, which capture both porous medium flow and the displacement. Lymph movement within the vessel is regulated by biological factors, specifically calcium ion ($\text {Ca}^{2+}$) concentration and NO production, which are included to modulate the pumping mechanism. Using this model, we quantify lymph flow and fluid exchange for both wild-type (WT) and diabetic vessels. Our findings illustrate how variations in NO production rate, Young’s modulus, and hydraulic conductivity impact leakage and pumping efficiency. The results demonstrate that reduced NO production in diabetic conditions leads to increased fluid leakage and altered pulsation frequency, while increased vessel stiffness further compromises lymphatic function.

## Introduction

**Fig. 1:**
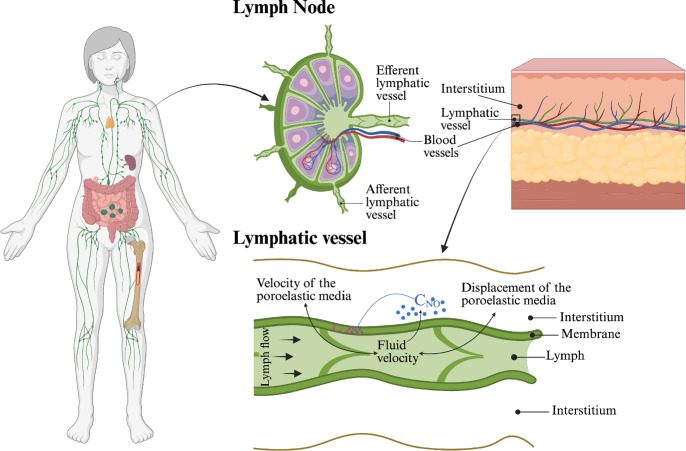
A model describing the interaction between the lymph flow, collecting vessel displacement, and flow and the displacement of the interstitium. The image presents a comprehensive depiction of the lymphatic system. The focus of this paper is on the modeling of the fluid exchange between a collecting lymph vessel and the interstitium (top right). The bottom panel describes the components taken into account in our model: Fluid velocity and the displacement of the intersititum, modeled as a poroelastic material, are coupled to the lymph velocity and pressure, and the displacement of the collecting vessel. The pumping dynamics is modulated by the concentration of nitric oxide (NO) and calcium ion $(\text {Ca}^{2+})$. *Created with BioRender.com*

The lymphatic system contains a network of vessels, nodes, and accessory organs crucial for maintaining the body’s interstitial fluid volume balance (see Fig. 1). Its function involves draining lymph from the interstitial space and returning it to the bloodstream (Nelson et al. 2014). Lymph is formed when the interstitial fluid enters the initial lymphatics (Li et al. 2023), and is then transported to the collecting lymphatics (Li et al. 2023). The collecting lymphatic vessels, surrounded by smooth muscle, transport lymph further to the lymph nodes (Stephens and Von Der Weid 2020) and actively propel it toward the venous circulation (Bazigou et al. 2014). The disfunction of lymphatic vessels has been linked to several metabolic diseases, including obesity, atherosclerosis, and type 2 diabetes. In type 2 diabetes, it was experimentally shown that due to the low bioavailability of nitric oxide (NO), the lymphatic vessels become leakier, exhibiting higher permeability (Scallan et al. 2015). The focus of this work is to model the lymph flow and fluid exchange with the interstitium, and to quantify how different components of this system are affected when the permeability and the NO concentration change in diabetic vessels. We develop a novel model of the lymphatic system which captures the interaction between lymph flow, vessel wall displacement, and the flow and the displacement of the surrounding tissue. We consider the lymph flow in a section of a collecting vessel, within a lymphangion, which is the segment between two valves. To model the lymph flow, we use the Stokes equations for an incompressible, viscous fluid, and to describe the vessel wall displacement, we use the Koiter shell model (Bukac et al. 2013). The interstitium is modeled as a poroelastic material, consisting of a porous medium flow phase and an elastic solid phase, and is described using the Biot model. To propel the lymph toward the lymph nodes, the collecting vessels exhibit periodic, phasic contractions primarily driven by the rhythmic action of smooth muscle cells.

**Fig. 2:**
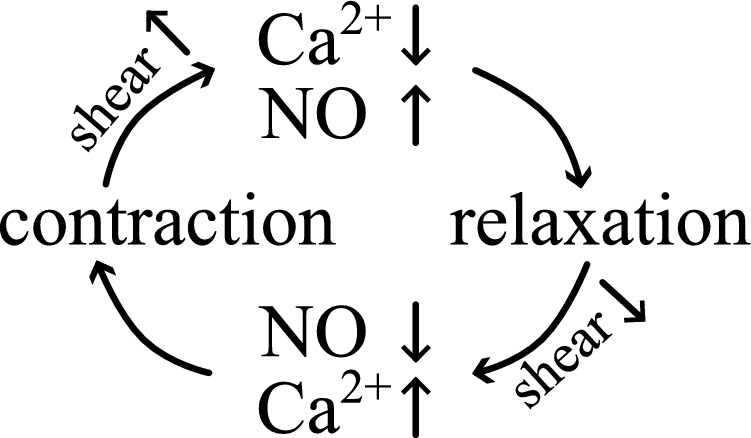
**Lymphatic pumping**. An increase in NO relaxes the vessel wall, increasing its diameter and drawing lymph into the lymphangion. As the vessel fills, shear stress and flow are reduced, leading to NO degradation. With NO inhibition removed, $\hbox {Ca}^{2+}$ influx triggers a contraction, which drives lymph forward and initiates a new pumping cycle (Kunert et al. 2015)

These contractions enable lymphatic vessels to provide pumping when needed, while valves remain open when flow can be driven by tissue pressure or gravity (Kunert et al. 2015; Mendoza and Schmid-Schonbein 2003). As described in Kunert et al. (2015), a pair of complementary mechanobiological oscillators is sufficient to regulate fluid transport within the lymphatic system. Those oscillators include calcium ion $(\text {Ca}^{2+})$ - mediated contractions, which are triggered by vessel stretch, and NO production in response to the resulting fluid shear stress, which lead to the local relaxation of lymphatic vessels (Kunert et al. 2015) (see Fig. 2).

To include the pumping effects regulated by mechanobiological oscillators in our model, we consider a system of coupled advection-diffusion equations describing the concentration of $\hbox {Ca}^{2+}$ and NO. Both $\hbox {Ca}^{2+}$ and NO contribute to the forcing acting on the vessel wall, which allows them to regulate the flow. The system of equations describing this interaction was introduced in Kunert et al. (2015); Baish et al. (2016); Li et al. (2019), however, a poroelastic description of the interstitium was not included in that work. Adding a poroelastic model for the interstitium allows us to study the fluid exchange with the collecting vessel, as well as the effects of the pumping disfunction on the lymph flow and the displacement of the vessel. In this work, we study the lymph flow in both a wild type (WT) vessel, and a diabetic vessel. By varying different parameters in our model, such as the NO production rate, permeability and Young’s modulus, we consider several cases of vessel wall dysfunction which occurs in type 2 diabetes. We quantify the main differences in the vessel wall displacement, which indicates pumping strength, the lymph flux across the vessel wall, which measures leakage, and the NO and $\hbox {Ca}^{2+}$ production, as the parameter values change. Our results demonstrate that increasing NO production alone is insufficient to restore lymphatic function; effective recovery requires normalization of both NO production and vessel wall permeability to healthy levels. We also find that pumping efficiency is strongly influenced by vessel wall stiffness. Since lymphatic vessels stiffen with age (Gashev and Chatterjee 2013), our model predicts that elderly patients with type 2 diabetes experience more pronounced pumping dysfunction compared to younger patients with more compliant vessels, in agreement with clinical observations by Shang et al. (2019).

The remainder of the paper is organized as follows. In Sect. 2, we introduce the mathematical model, present the variational formulation of the coupled problem, and describe the numerical discretization and computational setup. In Sect. 3, we present and discuss the computational results for both WT and diabetic vessels. Concluding remarks are given in Sect. 4.

1

## Materials and methods

### Mathematical model

**Fig. 3:**
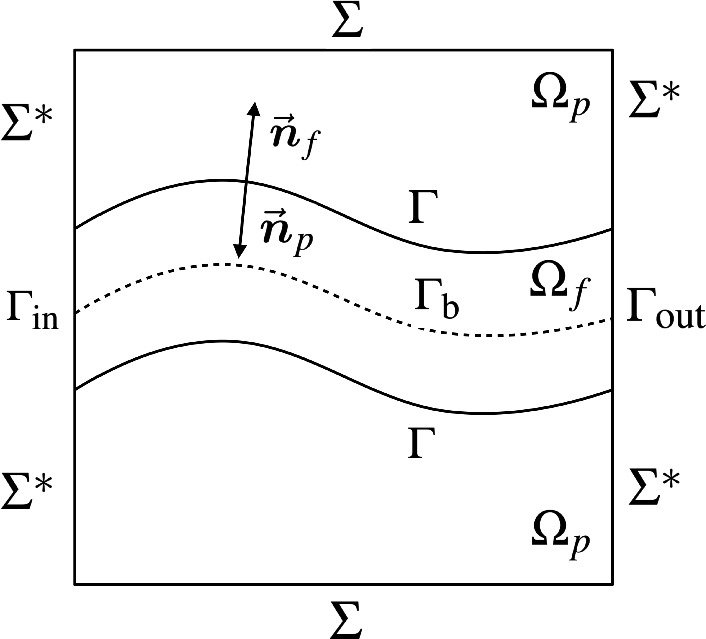
**Domains.** The fluid domain is represented by $\Omega _f$, the poroelastic structure domain by $\Omega _p$ and the thin structure domain by Γ. *Created with BioRender.com*

Let $\Omega \subset \mathbb {R}^2$ be an open bounded domain, consisting of a region filled by an incompressible, viscous fluid, denoted by $\Omega _f,$ and a region occupied by a poroelastic structure, denoted by $\Omega _p$. We assume that $\bar{\Omega } = \bar{\Omega }_f \cup \bar{\Omega }_p$ and $\Omega _f \cap \Omega _p = \emptyset$ (see Fig. 3).

2

The common interface between $\Omega _f$ and $\Omega _p$, denoted by Γ, represents a thin, elastic vessel wall. The midline of the fluid domain is denoted by $\Gamma _{\text {b}}$, and the external boundaries are denoted by $\Gamma _{\text {in}}$ and $\Gamma _{\text {out}}$. The external boundaries of the poroelastic domain are denoted by $\Sigma ^{*}$ and Σ. The outward unit normal vectors to $\partial \Omega _f$ and $\partial \Omega _p$ are denoted by $\vec {\boldsymbol{n}}_f$ and $\vec {\boldsymbol{n}}_p$, respectively, and the tangent vectors corresponding to $\vec {\boldsymbol{n}}_f$ are denoted by $\vec {\boldsymbol{\tau }}_{f}$. Next, we model the fluid flow, the poroelastic medium, and the elastic wall following established approaches commonly used in the literature (Kunštek et al. 2025; Oyekole and Bukac 2019; Bukac et al. 2015; Bukac 2016; Bukac et al. 2013), adapting them to suit the specifics of our problem.

We model the fluid flow using the time dependent Stokes equations, given by

1a $$\begin{aligned}&\rho _f \partial _t\vec {\boldsymbol{u}}= \nabla \cdot \boldsymbol{\sigma }_f&\text {in}\; \Omega _f , \end{aligned}$$

1b $$\begin{aligned}&\nabla \cdot \vec {\boldsymbol{u}}= 0&\text {in}\; \Omega _f , \end{aligned}$$

where $\rho _f$ is the fluid density, $\vec {\boldsymbol{u}}=(u_x, u_y)$ is the fluid velocity, where by *x* and *y* we denote the axial and the radial coordinates, respectively, $\boldsymbol{\sigma }_f= 2\mu _f\boldsymbol{D}(\vec {\boldsymbol{u}}) -p_f \textbf{I}$ is the fluid stress tensor, $\mu _f$ is the fluid viscosity, $\boldsymbol{D}(\vec {\boldsymbol{u}}) = (\nabla \vec {\boldsymbol{u}}+(\nabla \vec {\boldsymbol{u}})^T)/2$ is the strain rate tensor and $p_f$ is the fluid pressure. The flow is driven by the pressure drop imposed at the inflow and outflow boundaries. Assuming that the domain is axially symmetric, we model only the upper half of the domain, in which case we assign symmetry boundary conditions on $\Gamma _b,$ specified in (1e) and (1f) below. Assuming a rectangular domain, the boundary conditions are given by:

1c $$\begin{aligned}&\boldsymbol{\sigma }_f \vec {\boldsymbol{n}}_f = - 0.1 \boldsymbol{n}&\text {on}\; \Gamma _{\text {in}} , \end{aligned}$$

1d $$\begin{aligned}&\boldsymbol{\sigma }_f \vec {\boldsymbol{n}}_f =0&\text {on}\; \Gamma _{\text {out}} ,\end{aligned}$$

1e $$\begin{aligned}&u_y =0&\text {on}\; \Gamma _{\text {b}} ,\end{aligned}$$

1f $$\begin{aligned}&\partial _y u_x =0&\text {on}\; \Gamma _{\text {b}}. \end{aligned}$$

The poroelastic structure is described using the Biot model, given by

2a $$\begin{aligned}&\rho _p \partial _t\vec {\boldsymbol{\xi }}= \nabla \cdot \boldsymbol{\sigma }_p&\text {in}\; \Omega _p , \end{aligned}$$

2b $$\begin{aligned}&\boldsymbol{\kappa }^{-1} \vec {\boldsymbol{q}}= -\nabla p_p&\text {in}\; \Omega _p , \end{aligned}$$

2c $$\begin{aligned}&c_0 \partial _tp_p +\alpha \nabla \cdot \vec {\boldsymbol{\xi }}+ \nabla \cdot \vec {\boldsymbol{q}}= 0&\text {in}\; \Omega _p , \end{aligned}$$

where $\rho _p$ is the density of the solid material, $\vec {\boldsymbol{\eta }}$ is the displacement, and $\vec {\boldsymbol{\xi }}=\partial _t\vec {\boldsymbol{\eta }}$ is the structure velocity. The stress tensor $\boldsymbol{\sigma }_p$ is defined by

$$\begin{aligned} \boldsymbol{\sigma }_p(\vec {\boldsymbol{\eta }})=2\mu _s\boldsymbol{D}(\vec {\boldsymbol{\eta }})+\lambda _s(\nabla \cdot \vec {\boldsymbol{\eta }})\textbf{I}-\alpha p_p \textbf{I}, \end{aligned}$$

where $\mu _s$ and $\lambda _s$ are the material-dependent Lamé’s first and second parameters, α is the Biot-Willis coefficient, $\vec {\boldsymbol{q}}$ is the relative velocity of the fluid within the porous structure and $p_p$ is the fluid pressure in the poroelastic medium. The storage coefficient is denoted by $c_0$ and $\boldsymbol{\kappa }$ is the hydraulic conductivity. For the poroelastic structure, we prescribe the following boundary conditions:

2d $$\begin{aligned}&\boldsymbol{\sigma }_p \vec {\boldsymbol{n}}_p =0&\text {on}\; \Sigma ,\end{aligned}$$

2e $$\begin{aligned}&\eta _x=0, \; \vec {\boldsymbol{\tau }}\cdot \boldsymbol{\sigma }_p \vec {\boldsymbol{n}}_p =0&\text {on}\; \Sigma ^* ,\end{aligned}$$

2f $$\begin{aligned}&p_p =0&\text {on}\; \Sigma ,\end{aligned}$$

2g $$\begin{aligned}&\vec {\boldsymbol{q}}\cdot \vec {\boldsymbol{n}}_p =0&\text {on}\; \Sigma ^*. \end{aligned}$$

To model the collecting vessel wall, we use the Koiter shell model, given by Bukac et al. (2013):

3a $$\begin{aligned}&\rho _s h \partial _t\vec {\boldsymbol{w}}+ \boldsymbol{\mathcal {L}}\vec {\boldsymbol{d}}=\vec {\boldsymbol{f}}_d&\text {on}\; \Gamma , \end{aligned}$$

where $\rho _s$ is the structure density, *h* is the structure thickness, $\vec {\boldsymbol{d}}$ is the displacement, $\vec {\boldsymbol{w}}=\partial _t\vec {\boldsymbol{d}}$ is the structure velocity, $\boldsymbol{\mathcal {L}}$ is the linear differential operator derived from the elastic energy of the thin structure, and $\vec {\boldsymbol{f}}_d$ is the force applied to the elastic wall. Assuming axial symmetry, the Koiter shell model can be written component-wise as follows:

3b $$\begin{aligned}&\rho _s h \frac{\partial ^2 d_x}{\partial t^2} -C_2 \frac{\partial d_y}{\partial x}-C_3 \frac{\partial ^2 d_x}{\partial x ^2} =f_{d,x}&\text {on}\; \Gamma , \end{aligned}$$

3c $$\begin{aligned}&\rho _s h \frac{\partial ^2 d_y}{\partial t^2} +c_0 d_y-C_1 \frac{\partial ^2 d_y}{\partial x ^2} +C_2 \frac{\partial d_x}{\partial x}=f_{d,y}&\text {on}\; \Gamma , \end{aligned}$$

where $d_x$ denotes the axial displacement, $d_y$ denotes the radial displacement, and

$$ c_0=\frac{hE}{R^2(1-\nu ^2)}\left( 1+\frac{h^2}{12R^2}\right) , \, C_1=\frac{h^3}{6}\frac{E \nu }{R^2\left( 1-\nu ^2\right) }, $$

$$ C_2=\frac{h}{R}\frac{E \nu }{1-\nu ^2} , \, C_3=\frac{hE}{1-\nu ^2}. $$

We assume that the thin structure is fixed at the edges:

3d $$\begin{aligned}&\vec {\boldsymbol{d}}= 0&\text {on}\; \partial \Gamma . \end{aligned}$$

Next, we introduce the models for the concentration of the $\text {Ca}^{2+}$ and NO. Similar as in Kunert et al. (2015); Li et al. (2019), we describe the $\text {Ca}^{2+}$ dynamics as follows:

4a $$\begin{aligned} \partial _tC_{\text {Ca}}&=D_{\text {Ca}} \Delta C_{\text {Ca}} -K^-_{\text {Ca}} \left( 1+K_{\text {Ca,NO}}C_{\text {NO}}\right) C_{\text {Ca}} \lambda + K^+_{\text {Ca}} \lambda \nonumber \\&\quad + K^+_{\text {Ca}} \lambda \left( \frac{R-R_l}{R_{\text {Ca}}-R_l}\right) ^{11} +10 \lambda K _ \delta ^+ \delta \! \! \uparrow \left( C_{\text {th}},C_{\text {Ca}}\right)&\text {on}\; \Gamma , \end{aligned}$$

where $C_{\text {Ca}}$ is the concentration of $\text {Ca}^{2+}$ and $D_{\text {Ca}}$ represents the effective diffusion coefficient of $\text {Ca}^{2+}$ spreading from one cell to the neighboring cells along the vessel wall. The decay rate of $\text {Ca}^{2+}$, the production rate of $\text {Ca}^{2+}$, and the rate constant for NO inhibition of $\text {Ca}^{2+}$ are denoted by $K^-_{\text {Ca}}$, $K^+_{\text {Ca}}$, and $K_{\text {Ca,NO}}$, respectively. The wall’s elastic response is taken into account by $K^+_{\text {Ca}} \left( \frac{R-R_l}{R_{\text {Ca}}-R_l}\right) ^{11}$, where *R* is the current radius of the vessel, $R_l$ is the limiting minimum radius of the vessel, $R_{\text {Ca}}$ is the threshold radius for $\text {Ca}^{2+}$ channel sensitization, and the 11th power term was chosen to generate a rapidly increasing force (Baish et al. 2016; Li et al. 2019) corresponding to the dramatic increase of $\hbox {Ca}^{2+}$ as the radius of the vessel exceeds a reference radius. However, this value was chosen in Li et al. (2019) arbitrarily, and it was mentioned that other similar functions could be substituted without significant consequence. Coefficient λ is the chemical reaction rate constant, and $\delta \! \! \uparrow$ is an asymmetric Kronecker δ-function defined to be equal to 1 when $C_{\text {Ca}}$ exceeds the threshold $C_{\text {th}}$ by increasing from below, and 0 otherwise. This is because when $C_{\text {Ca}}$ reaches the threshold $C_{\text {th}}$, it triggers action potentials mediated by voltage gated and calcium-induced calcium channels. $K^+_{\delta }$ represents a sharp increase in $C_{\text {Ca}}$ when it exceeds $C_{\text {th}}$. All parameter values can be found in Table 1. It was assumed in Kunert et al. (2015) that the calcium enters the cytosol at a baseline rate, which was set equal to the decay rate. This ensures a stable baseline calcium concentration in the model. For this problem, as in Li et al. (2019), we prescribe the following boundary conditions:

4b $$\begin{aligned}&\nabla C_{\text {Ca}} \cdot \vec {\boldsymbol{n}}= 0&\text {on}\; \partial \Gamma . \end{aligned}$$

The model we use is taken from Kunert et al. (2015); Li et al. (2019).

In response to the changes in the shear stress, NO is rapidly produced by the endothelium and quickly degraded (Zhao et al. 2015). To allow the NO to convect and diffuse in both the fluid and tissue spaces, we describe the NO dynamics using the following equation (Li et al. 2019):

5a $$\begin{aligned}&\partial _tC_{\text {NO}}= D_{\text {NO}} \Delta C_{\text {NO}} - \vec {\boldsymbol{u}}\cdot \nabla C_{\text {NO}} + \left( - K_{\text {NO}}^- C_{\text {NO}} + K_{\text {NO}}^+ \bigg | \frac{\partial u_x }{\partial y} \bigg | \right) \lambda&\text {in}\; \Omega , \end{aligned}$$

where $C_{\text {NO}}$ is the concentration of NO, $D_{\text {NO}}$ is the diffusion coefficient, $K_{\text {NO}}^-$ is the decay rate, and $K_{\text {NO}}^+$ is the production coefficient. The production of NO is proportional to the stress component $\left| \displaystyle \frac{\partial u_x }{\partial y} \right|$ where $u_x$ is the fluid velocity along the tangential direction of the wall surface. Similar as in Li et al. (2019), the following boundary conditions are prescribed:

5b $$\begin{aligned}&\nabla C_{\text {NO}} \cdot \vec {\boldsymbol{n}}= 0&\text {on}\; \Sigma . \end{aligned}$$

5c $$\begin{aligned}&C_{\text {NO}} = 0&\text {on}\; \Sigma ^* \cup \Sigma _{in} \cup \Sigma _{out}. \end{aligned}$$

To couple the fluid, thin elastic structure, and the poroelastic material, we prescribe the mass conservation, the slip condition with a slip rate γ>0, the momentum conservation, the balance of contact forces, and the continuity of displacement on $\Gamma \times (0,T)$:

6a $$\begin{aligned}&\text {Mass conservation:}&\vec {\boldsymbol{n}}_f\cdot \vec {\boldsymbol{u}}= \vec {\boldsymbol{n}}_f\cdot (\vec {\boldsymbol{\xi }}+ \vec {\boldsymbol{q}}),\end{aligned}$$

6b $$\begin{aligned}&\text {Slip condition: }&\vec {\boldsymbol{\tau }}_{f} \cdot \boldsymbol{\sigma }_f \vec {\boldsymbol{n}}_f = -\gamma (\vec {\boldsymbol{u}}- \vec {\boldsymbol{\xi }})\cdot \vec {\boldsymbol{\tau }}_{f},\end{aligned}$$

6c $$\begin{aligned}&\text {Momentum conservation: }&\vec {\boldsymbol{n}}_f \cdot \boldsymbol{\sigma }_f \vec {\boldsymbol{n}}_f = -p_p, \end{aligned}$$

6d $$\begin{aligned}&\text {Balance of contact forces: }&\boldsymbol{\sigma }_f \vec {\boldsymbol{n}}_f \cdot \vec {\boldsymbol{n}}_f = \boldsymbol{\sigma }_p \vec {\boldsymbol{n}}_f \cdot \vec {\boldsymbol{n}}_f + \vec {\boldsymbol{f}}-\vec {\boldsymbol{f}}_d \cdot \vec {\boldsymbol{n}}_f ,\end{aligned}$$

6e $$\begin{aligned} &  \boldsymbol{\sigma }_f \vec {\boldsymbol{n}}_f \cdot \vec {\boldsymbol{\tau }}_{f} = \boldsymbol{\sigma }_p \vec {\boldsymbol{n}}_f \cdot \vec {\boldsymbol{\tau }}_{f} -\vec {\boldsymbol{f}}_d \cdot \vec {\boldsymbol{\tau }}_{f}, \end{aligned}$$

6f $$\begin{aligned}&\text {Continuity of displacement: }&\vec {\boldsymbol{d}}= \vec {\boldsymbol{\eta }}. \end{aligned}$$

The forcing term $\vec {\boldsymbol{f}}$ that appears in (6d), acting on the vessel wall, is given by Kunert et al. (2015); Li et al. (2019):

7 $$\begin{aligned}&\vec {\boldsymbol{f}}= K_M \left( \frac{C_{\text {Ca}}}{1+C_{\text {Ca}}}\right) \left( \frac{2R}{R_{\text {Ca}}+R}\right) \left( \frac{1}{1+K_{\text {NO}}C_{\text {NO}}}\right) , \end{aligned}$$

where $K_M$ is a constant which regulates the strength of the contracting force, $K_{\text {NO}}$ is a coefficient which controls the strength of the NO inhibition, and *R* is the current radius of the vessel which is relative to the reference value $R_{\text {Ca}}$.

In our model, the valves are taken into account through the pumping dynamics regulated by the mechanobiological oscillators described in (4a) and (5a). In particular, the increase in production of NO relaxes the wall, as in that case the lymph is pulled into the lymphangion through the open upstream valve. The increase in production of $\hbox {Ca}^{2+}$ contracts the wall and reduces the flow. The ability of the oscillator to affect the flow is achieved by imposing a pressure drop at the inlet and outlet sections. Backflow suppression is thus achieved implicitly through the interplay between the mechanobiological oscillator and the prescribed pressure drop, rather than through an explicit geometric or constitutive valve representation. This approach is appropriate for the targeted physiological regime of a single lymphangion where the focus is on transmural fluid exchange rather than detailed valve leaflet mechanics.

### Variational formulation of the fluid-thin structure-poroelastic structure interaction problem

For an open set *X*, where *X* may be $\Omega _f$, $\Omega _p$ or Γ, we denote by $(\cdot ,\cdot )_X$ and $\left\| \cdot \right\| _X$ the standard $L^2(X)$ inner-product and norm, respectively. We define the following Sobolev spaces:

$$\begin{aligned}&\boldsymbol{V}^f = \{\vec {\boldsymbol{u}}\in (H^1(\Omega _f))^2\;|\; u_y=0\hspace{5.0pt}\text {on}\hspace{5.0pt}\Gamma _b\}, \\&\boldsymbol{V}^s = \{\vec {\boldsymbol{\varphi }}\in (H^1(\Omega _p))^2\;|\; \vec {\boldsymbol{\varphi }}=0\hspace{5.0pt}\text {on}\hspace{5.0pt}\Sigma ^*\},\\&Q^f = L^2(\Omega _f),\\&Q^p = L^2(\Omega _p),\\&\boldsymbol{V}^d = \{\vec {\boldsymbol{q}}\in H(\text {div},\Omega _p)\;|\; \vec {\boldsymbol{q}}\cdot \vec {\boldsymbol{n}}_p=0\hspace{5.0pt}\text {on}\hspace{5.0pt}\Sigma ^* \} \\ \end{aligned}$$

where $$H(\text {div},\Omega _p)=\{\vec {\boldsymbol{q}}\in L^2(\Omega _p): \nabla \cdot \vec {\boldsymbol{q}}\in L^2(\Omega _p)\}$$.

#### Definition 1

We say that $(\vec {\boldsymbol{u}},p,\vec {\boldsymbol{\eta }},\vec {\boldsymbol{q}},p_p) \in \boldsymbol{V}^f \times {Q^f} \times \boldsymbol{V}^s \times \boldsymbol{V}^d\times {Q^p}$ is a weak solution to the fluid-thin structure-poroelastic structure interaction problem if $\vec {\boldsymbol{u}}\cdot \vec {\boldsymbol{n}}_f= (\vec {\boldsymbol{\xi }}+ \vec {\boldsymbol{q}}) \cdot \vec {\boldsymbol{n}}_f$ on Γ, and the following equality holds:

$$\begin{aligned}&\rho _f(\partial _t\vec {\boldsymbol{u}},\vec {\boldsymbol{v}})_{\Omega _f} + 2\mu _f(\boldsymbol{D}(\vec {\boldsymbol{u}}),\boldsymbol{D}(\vec {\boldsymbol{v}}))_{\Omega _f} - (p,\nabla \cdot \vec {\boldsymbol{v}})_{\Omega _f}+(\nabla \cdot \vec {\boldsymbol{u}},\psi )_{\Omega _f} \\&\qquad +\rho _p(\partial _t\vec {\boldsymbol{\xi }},\vec {\boldsymbol{\varphi }})_{\Omega _p} + 2\mu _s(\boldsymbol{D}(\vec {\boldsymbol{\eta }}),\boldsymbol{D}(\vec {\boldsymbol{\varphi }}))_{\Omega _p} + \lambda _s(\nabla \cdot \vec {\boldsymbol{\eta }}, \nabla \cdot \vec {\boldsymbol{\varphi }})_{\Omega _p}-\alpha (\nabla \cdot \vec {\boldsymbol{\varphi }}, p_p)_{\Omega _p}\\&\qquad +(\boldsymbol{\kappa }^{-1} \vec {\boldsymbol{q}},\vec {\boldsymbol{r}})_{\Omega _p}-(p_p,\nabla \cdot \vec {\boldsymbol{r}})_{\Omega _p} +c_0 (\partial _tp_p,s)_{\Omega _p} +\alpha (\nabla \cdot \vec {\boldsymbol{\xi }},s)_{\Omega _p} + (s, \nabla \cdot \vec {\boldsymbol{q}})_{\Omega _p} \\&\qquad + \rho _s h( \partial _t\vec {\boldsymbol{\xi }},\vec {\boldsymbol{\varphi }})_\Gamma + (\boldsymbol{\mathcal {L}}\vec {\boldsymbol{\eta }},\vec {\boldsymbol{\varphi }})_\Gamma + \gamma \left( (\vec {\boldsymbol{u}}- \vec {\boldsymbol{\xi }})\cdot \vec {\boldsymbol{\tau }}_{f},(\vec {\boldsymbol{v}}-\vec {\boldsymbol{\varphi }})\cdot \vec {\boldsymbol{\tau }}_{f} \right) _{\Gamma } \\&\quad =\Bigl (\vec {\boldsymbol{f}},\vec {\boldsymbol{\varphi }}\cdot \vec {\boldsymbol{n}}_f \Bigr )_\Gamma \end{aligned}$$

for all $(\vec {\boldsymbol{v}},\psi ,\vec {\boldsymbol{\varphi }}, \vec {\boldsymbol{r}}, s)\in \boldsymbol{V}^f \times {Q^f}\times \boldsymbol{V}^s \times \boldsymbol{V}^d\times {Q^p}$ such that $\vec {\boldsymbol{n}}_f\cdot \vec {\boldsymbol{v}}= \vec {\boldsymbol{n}}_f\cdot (\vec {\boldsymbol{\varphi }}+ \vec {\boldsymbol{r}})$ on Γ.

### Numerical method

Let $t^n = n \tau$ for n=0,…,N, where τ denotes the time step. For a function $\boldsymbol{v},$ we introduce the following notation for the discrete time derivative:

$$ d_t \boldsymbol{v}^{n} = \frac{\boldsymbol{v}^{n}-\boldsymbol{v}^{n-1}}{\tau }. $$

To discretize the problem in time, the governing equations are written as a first-order system, and the backward Euler method is applied. While this approach introduces numerical dissipation, its effect is less significant here, as physical dissipation from the viscous fluid is already present in the coupled system. This approach is expected to be unconditionally stable and first-order accurate in time. For spatial discretization, we use the finite element method, as in Bukac et al. (2015). Let $T_h^f$ and $T_h^p$ be triangular, quasi-uniform meshes on domains $\Omega _f$ and $\Omega _p$, respectively, so that the ratio of the diameter of any element to the diameter of its largest inscribed ball is uniformly bounded (Brenner and Scott 2008). We select $\mathbb {P}^2$ elements for velocities and $\mathbb {P}^1$ elements for the pressure, as well as the NO and $\text {Ca}^{2+}$ concentrations. The discrete spaces are subsets of their continuous counterparts:

$$\begin{aligned}&\boldsymbol{V}^f_h\subset \boldsymbol{V}^f, &  \boldsymbol{V}^s_h\subset \boldsymbol{V}^s,\\&\boldsymbol{V}^d_h\subset \boldsymbol{V}^d, &  Q^p_h \subset Q^p, \\&Q^f_h\subset Q^f, &  { Q^{\text {Ca}}_h} \subset H_0^1(\Gamma ), \\&{ Q^{\text {NO}}_h }\subset H_0^1(\Omega ). &  \end{aligned}$$

Since we use the dual-mixed formulation in the Biot problem, (6a) is a kinematic condition, which is difficult to impose in the test spaces. Therefore, we use Nitsche’s method to weakly enforce the continuity of normal flux at the discrete level (Bukac et al. 2015; Hansbo 2005). We note that the weak formulation in Definition 1 is obtained assuming (6a) holds for both test and trial functions. In order to apply Nitsche’s method, we first start from the combined the interface terms given by:

$$\begin{aligned}&I_\Gamma :=\int _{\Gamma }\left( \boldsymbol{\sigma }_{f,h}\vec {\boldsymbol{n}}_f \cdot \vec {\boldsymbol{v}}_h-\boldsymbol{\sigma }_{p,h}\vec {\boldsymbol{n}}_f\cdot \vec {\boldsymbol{\varphi }}_h+p_{p,h}\vec {\boldsymbol{r}}_{h}\cdot \vec {\boldsymbol{n}}_f+\vec {\boldsymbol{f}}_{d,h}\vec {\boldsymbol{b}}_h\right) . \end{aligned}$$

Using Nitsche’s method, we enforce the interface conditions (6a)-(6f) as follows:

$$\begin{aligned} I_\Gamma&=\int _{\Gamma }\vec {\boldsymbol{n}}_f\cdot \boldsymbol{\sigma }_{f,h}\vec {\boldsymbol{n}}_f\left( \vec {\boldsymbol{v}}_h-\vec {\boldsymbol{r}}_h-\vec {\boldsymbol{\varphi }}_h\right) \cdot \vec {\boldsymbol{n}}_f +\int _{\Gamma } \vec {\boldsymbol{\tau }}_{f}\cdot \boldsymbol{\sigma }_{f,h}\vec {\boldsymbol{n}}_f\left( \vec {\boldsymbol{v}}_h-\vec {\boldsymbol{\varphi }}_h\right) \cdot \vec {\boldsymbol{\tau }}_{f}\\&\quad +\int _{\Gamma }\vec {\boldsymbol{f}}_h\vec {\boldsymbol{\varphi }}_h\cdot \vec {\boldsymbol{n}}_f. \end{aligned}$$

The kinematic condition (6a) is then imposed weakly, and following terms are added to the weak formulation:

$$\begin{aligned}&\int _{\Gamma }\gamma _f\mu _f h^{-1}\left( \vec {\boldsymbol{u}}_h-\vec {\boldsymbol{q}}_h-\vec {\boldsymbol{\xi }}_h\right) \cdot \vec {\boldsymbol{n}}_f\left( \vec {\boldsymbol{v}}_h-\vec {\boldsymbol{r}}_h-\vec {\boldsymbol{\varphi }}_h\right) \cdot \vec {\boldsymbol{n}}_f\\&-\int _{\Gamma } \vec {\boldsymbol{n}}_f\cdot \boldsymbol{\sigma }_{f,h}(\zeta \vec {\boldsymbol{v}}_h, -\psi _h) \vec {\boldsymbol{n}}_f \left( \vec {\boldsymbol{u}}_h-\vec {\boldsymbol{q}}_h-\vec {\boldsymbol{\xi }}_h\right) \cdot \vec {\boldsymbol{n}}_f, \end{aligned}$$

where $\gamma _f>0$ denotes a penalty parameter and $\zeta \in \{-1,0,1 \}$ determines if we adopt a symmetric, incomplete, or skew-symmetric formulation.

Therefore, the fully discrete coupled fluid-elastic structure-poroelastic structure interaction problem is given by:

Find $(\vec {\boldsymbol{u}}_h^n,p_h^n,\vec {\boldsymbol{\eta }}_h^n, p_{p,h}^n,\vec {\boldsymbol{q}}_h^n) \in \boldsymbol{V}_h \times {Q^f_h}\times \boldsymbol{V}^s_h\times {Q^p_h}\times \boldsymbol{V}^d_h$ where $\vec {\boldsymbol{\xi }}_h^n=d_t \vec {\boldsymbol{\eta }}_h^n$ such that for any $(\vec {\boldsymbol{v}}_h, \psi _h,\vec {\boldsymbol{\varphi }}_h, \vec {\boldsymbol{r}}_h, s_h) \in \boldsymbol{V}^v_h \times {Q^f_h}\times \boldsymbol{V}^s_h\times {Q^p_h}\times \boldsymbol{V}^d_h$ we have

$$\begin{aligned}&\rho _f(d_\tau \vec {\boldsymbol{u}}_h^n,\vec {\boldsymbol{v}}_h)_{\Omega _f} + 2\mu _f(\boldsymbol{D}(\vec {\boldsymbol{u}}_h^n),\boldsymbol{D}(\vec {\boldsymbol{v}}_h))_{\Omega _f} - (p_h^n,\nabla \cdot \vec {\boldsymbol{v}}_h)_{\Omega _f}+(\nabla \cdot \vec {\boldsymbol{u}}_h^n,\psi _h)_{\Omega _f} \\&+\rho _p(d_\tau \vec {\boldsymbol{\xi }}_h^n,\vec {\boldsymbol{\varphi }}_h)_{\Omega _p} + 2\mu _s(\boldsymbol{D}(\vec {\boldsymbol{\eta }}_h^n),\boldsymbol{D}(\vec {\boldsymbol{\varphi }}_h))_{\Omega _p} + \lambda _s(\nabla \cdot \vec {\boldsymbol{\eta }}_h^n, \nabla \cdot \vec {\boldsymbol{\varphi }}_h)_{\Omega _p} \\&-\alpha (p_{p,h}^n,\nabla \cdot \vec {\boldsymbol{\varphi }}_h)_{\Omega _p} +(\boldsymbol{\kappa }^{-1} \vec {\boldsymbol{q}}_h^n,\vec {\boldsymbol{r}}_h)_{\Omega _p}-(p_{p,h}^n,\nabla \cdot \vec {\boldsymbol{r}}_h)_{\Omega _p}\\&+c_0(\partial _tp_{p,h}^n,s_h)_{\Omega _p} +\alpha (\nabla \cdot \vec {\boldsymbol{\xi }}_h^n,s_h)_{\Omega _p} + (\nabla \cdot \vec {\boldsymbol{q}}_h^n, s_h)_{\Omega _p}\\&+\rho _s h ( \partial _t\vec {\boldsymbol{\xi }}_h^n,\vec {\boldsymbol{\varphi }}_h)_\Gamma + (\boldsymbol{\mathcal {L}}\vec {\boldsymbol{\eta }}_h^n,\vec {\boldsymbol{\varphi }}_h)_\Gamma + \gamma \left( (\vec {\boldsymbol{u}}_h^n - \vec {\boldsymbol{\xi }}_h^n)\cdot \vec {\boldsymbol{\tau }}_{f},(\vec {\boldsymbol{v}}_h-\vec {\boldsymbol{\varphi }}_h )\cdot \vec {\boldsymbol{\tau }}_{f} \right) _{\Gamma } \\&+\left( \gamma _f\mu _f h^{-1}\left( \vec {\boldsymbol{u}}_h^{n}-\vec {\boldsymbol{q}}_h^{n}-\vec {\boldsymbol{\xi }}_h^{n} \right) \cdot \vec {\boldsymbol{n}}_f, \left( \vec {\boldsymbol{v}}_h-\vec {\boldsymbol{r}}_h-\vec {\boldsymbol{\varphi }}_h\right) \cdot \vec {\boldsymbol{n}}_f \right) _{\Gamma } \\&-\left( \vec {\boldsymbol{n}}_f\cdot \boldsymbol{\sigma }_{f,h}(\zeta \vec {\boldsymbol{v}}_h, -\psi _h) \vec {\boldsymbol{n}}_f, \left( \vec {\boldsymbol{u}}_h^{n}-\vec {\boldsymbol{q}}_h^{n}-\vec {\boldsymbol{\xi }}_h^{n}\right) \cdot \vec {\boldsymbol{n}}_f \right) _{\Gamma } \\&=\left( \vec {\boldsymbol{n}}_f\cdot \boldsymbol{\sigma }_{f,h}^{n}\vec {\boldsymbol{n}}_f, \left( \vec {\boldsymbol{v}}_h-\vec {\boldsymbol{r}}_h-\vec {\boldsymbol{\varphi }}_h\right) \cdot \vec {\boldsymbol{n}}_f \right) _{\Gamma } +\left( \vec {\boldsymbol{f}}_h^{n-1} \vec {\boldsymbol{\varphi }}_h\cdot \vec {\boldsymbol{n}}_f \right) _{\Gamma }. \end{aligned}$$

The fully discrete problem for the NO concentration dynamics is given as follows: Find $C_{\text {NO},h}^n\in { Q^{\text {NO}}_h}$ such that for any $w_{\text {NO,h} }\in {Q^{\text {NO}}_h}$ we have

$$\begin{aligned}&\left( d_\tau C_{\text {NO},h}^n, w_{\text {NO},h}\right) _{\Omega } + \lambda \left( K_{\text {NO}}^- C_{\text {NO},h}^n,w_{\text {NO},h} \right) _{\Omega } - \lambda \left( K_{\text {NO}}^+ \bigg | \frac{\partial u_x^n }{\partial y} \bigg | ,w_{\text {NO},h} \right) _{\Omega } \\&\quad \quad + \left( \vec {\boldsymbol{u}}_h^n \cdot \nabla C_{\text {NO},h}^n, w_{\text {NO},h} \right) _{\Omega } +\left( D_{\text {NO}}\nabla C_{\text {NO},h}^n,\nabla w_{\text {NO},h}\right) _{\Omega } = 0. \end{aligned}$$

Finally, the fully discrete problem for the calcium dynamics is given by: Find $C_{\text {Ca},h}^n\in { Q^{\text {Ca}}_h }$ such that for any $w_{\text {Ca},h} \in { Q^{\text {Ca}}_h }$ we have

$$\begin{aligned}&\left( d_\tau C_{\text {Ca},h}^n,w_{\text {Ca},h} \right) _{\Gamma } +\lambda \left( K^-_{\text {Ca}} \left( 1+K_{\text {Ca,NO}}C_{\text {NO},h}^n\right) C_{\text {Ca},h}^n,w_{\text {Ca},h} \right) _{\Gamma } - \lambda \left( K^+_{\text {Ca}},w_{\text {Ca},h} \right) _{\Gamma } \\&\quad \quad - \lambda \left( K^+_{\text {Ca}} \left( \frac{R^n-R_l}{R_{\text {Ca}}-R_l}\right) ^{11},w_{\text {Ca},h} \right) _{\Gamma } -10 \lambda \left( K_\delta ^+ \delta \! \! \uparrow \left( C_{\text {th}},C_{\text {Ca}}\right) , w_{\text {Ca},h} \right) _{\Gamma } \\&\quad \quad + D_{\text {Ca}}\left( \nabla C_{\text {Ca},h}^n,\nabla w_{\text {Ca},h} \right) _{\Gamma } =0. \end{aligned}$$

### Computational setup and simulation parameters

In this section, we summarize the domain geometry and the parameters used in our study. In addition to the WT simulations, we will also consider different diabetic cases. The simulation parameters that are the same for all the cases considered in this study are given in Table 1.

**Table 1:** Simulation parameters

Parameter	Value	Units	Definition	Reference
*R*	0.006	$\text {cm}$	Radius of the vessel	(Kuan et al. 2015; Bertram et al. 2018)
*L*	0.08	$\text {cm}$	Length	
$\rho _F$	1	$\text {g/} \text {cm}^3$	Fluid density	Li et al. (2019)
$\mu _F$	0.01	$\text {cm}^2 \text {/s}$	Fluid viscosity	Li et al. (2019)
*h*	0.0002	$\text {cm}$	Membrane thickness	
*H*	0.0025	$\text {cm}$	Tissue thickness	
$\rho _p$	1	$\text {g/} \text {cm}^3$	Tissue density	
κ	$\left( 10^{-11}-10^{-8} \right)$	$\text {cm}^3 \text {s}/\text {g}$	Hydraulic conductivity of tissue	Li et al. (2023)
*E*	$(10^4-8 \cdot 10^5)$	$\text {dyn/} \text {cm}^2$	Young’s modulus	Li et al. (2023); Koudehi et al. (2023)
ν	0.45(0.42-0.49)	–	Poisson’s ratio	Li et al. (2023)
$\mu _s$	$\text {E}\nu / [2(1+\nu )]$	$\text {dyn/} \text {cm}^2$	Shear modulus	Li et al. (2023)
$\lambda _s$	$\text {E}\nu / [(1+\nu )(1-2\nu )]$	$\text {dyn/} \text {cm}^2$	Lame’s first parameter	Li et al. (2023)
$$c_0$$	$$ (0- 10^{-5})$$	–	Storage coefficient	
α	1(0.95-1)	–	Biot coefficient	Li et al. (2023)
$D_{\text {NO}}$	$1.2 \cdot 10^{-4}$	$\text {cm}^2 \text {/s}$	NO diffusivity	Li et al. (2019)
$K_{\text {NO}}^-$	20	$\text {s}^{-1}$	NO degradation rate constant	
$K_{\text {NO}}^+$	(50-200)	$\text {s}^{-1}$	NO production rate constant	Scallan et al. (2015); Li et al. (2019)
$D_{\text {Ca}}$	$6.5 \cdot 10^{-6}$	$\text {cm}^2 \text {/s}$	$\text {Ca}^{2+}$ signal propagation rate	Li et al. (2019)
$K_{\text {Ca}}^-$	37.6	$\text {s}^{-1}$	$\text {Ca}^{2+}$ degradation rate constant	Li et al. (2019)
$K_{\text {Ca}}^+$	1.2	$\text {s}^{-1}$	$\text {Ca}^{2+}$ production rate constant	Li et al. (2019)
$K_\delta ^+$	4000	$\text {s}^{-1}$	$\text {Ca}^{2+}$ production rate constant	
$C_{\text {th}}$	0.015	–	$\text {Ca}^{2+}$ threshold	Li et al. (2019)
$R_{\text {Ca}}$	$\text {R}_{0}$	$\text {cm}$	Threshold radius for $\text {Ca}^{2+}$ channel sensitization	Li et al. (2019)
$K_{\text {Ca,NO}}$	5.3	–	Rate constant for NO inhibition of $\text {Ca}^{2+}$	Li et al. (2019)
λ	0.03	–	Chemical reaction rate constant	Li et al. (2019)
$K_\text {M}$	200	$\text {dyn}$	Force constant for $\text {Ca}^{2+}$	
$K_\text {NO}$	1	–	NO inhibition of force	Li et al. (2019)
$R_\text {l}$	0.003	$\text {cm}$	Limit radius	Li et al. (2019)
$$ R_0$$	*R*	$$ \text {cm}$$	Rest radius of the vessel	Li et al. (2019)

Type 2 diabetes is characterized by the impaired NO production, which was shown to lower the permeability of diabetic lymphatics (Scallan et al. 2015). Hence, we use the hydraulic conductivity of $\boldsymbol{\kappa } = 2 \cdot 10^{-10}\,\text {cm}^3\text {s/g}$ for the WT, and the hydraulic conductivity of $\boldsymbol{\kappa } = 2.16 \cdot 10^{-8}\,\text {cm}^3\text {s/g}$ for diabetic vessels, consistent with the measurements from Scallan et al. (2015). To account for the impaired NO production, we consider three cases where we vary the NO production rate, as described in Table 2.

**Table 2:** Parameter values for different cases considered in this study

Parameter	Wild Type	Diabetes 1	Diabetes 2	Diabetes 3	Diabetes 4	Units
κ	$2\cdot 10^{-10}$	$2.16\cdot 10^{-8}$	$2.16\cdot 10^{-8}$	$2.16\cdot 10^{-8}$	$2.16\cdot 10^{-8}$	$\text {cm}^3 \text {s}/\text {g}$
*E*	$10^4$	$10^4$	$10^4$	$10^4$	$10^4$	$\text {dyn/} \text {cm}^2$
$K_{\text {NO}}^+$	200	150	100	50	200	$\text {s}^{-1}$

For one of the diabetic cases (Diabetes 2), we also study the effects of the vessel stiffness by changing the value of Young’s modulus, and the effects of the storage coefficient $c_0$. The storage coefficient is known to be ‘close to zero’, as the tissue is nearly incompressible, but it is difficult to measure it experimentally. Hence, we study the sensitivity of the solutions to the values of this parameter. Finally, in order to determine whether it is enough to increase only the NO production rate in order to restore the normal function of diabetic vessels, we consider the case Diabetes 4, where the NO production rate is the same as in WT, but the permeability still has a larger value, as in the other diabetic cases.

**Fig. 4:**
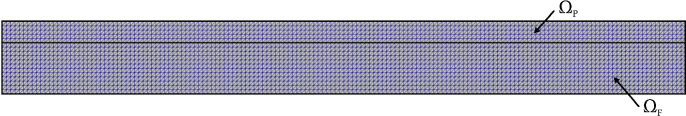
The computational mesh used in numerical simulations

For the numerical implementation of this problem, we use the finite element solver FreeFem++ (Hecht 2012). Due to the axial symmetry, we consider a 2D model. The computational mesh, shown in Fig. 4, consists of 3840 elements in the fluid region and 1600 elements in the poroelastic region.

The penalty parameter in Nitsche’s method is set to $\gamma _f = 2000$, as our computations showed that this value is large enough so that the kinematic condition is sufficiently satisfied. Furthermore, we adopt the symmetric formulation, in which case ζ=-1. We solve the fluid-poroelastic structure interaction problem using a time step of $\Delta t=10^{-3}$ and a total simulation duration of T=8 s.

## Results and Discussion

We present computational results for a WT collecting lymphatic vessel and several diabetic cases, as defined in Sect. 2.4. We begin by validating the baseline model. Table 3 compares key WT outputs, including contraction frequency, maximum diameter change, and average flow rate, against experimental measurements and published simulations, showing agreement within the ranges reported in the literature.

**Table 3:** Comparison of key simulation outputs with literature and experimental values

Quantity	WT simulation	Literature/clinical range	Reference
Contraction frequency ($\hbox {min}^{-1}$)	∼25	1–38	Li et al. (2019); Gashev et al. (2002); Kunert et al. (2015); Liao et al. (2011)
Max. diameter change (%)	∼11	10–40	Baish et al. (2016); Gashev et al. (2002); Liao et al. (2011); Akl et al. (2011); Baish et al. (2016)
Average flowrate in the lumen ($\hbox {cm}^3$/s)	$9.56 \cdot 10^{-6}$	$1.7 \cdot 10^{-6}-5.5 \cdot 10^{-4}$	Rahbar et al. (2014); Sarimollaoglu et al. (2018)

The subsequent subsections examine the effects of NO production rate, Young’s modulus, storage coefficient, and hydraulic conductivity.

### The effects of the NO production rate

In our simulations, we investigate how different NO production rates affect the NO and $\text {Ca}^{2+}$ concentrations in collecting lymphatic vessels, as well as the transmural flow and vessel wall displacement. All concentrations in the model are dimensionless, obtained by normalizing by a reference concentration taken to be unity. The parameters used in these simulations are given in Table 2 under Wild Type and Diabetes 1, 2, and 3.

**Fig. 5:**
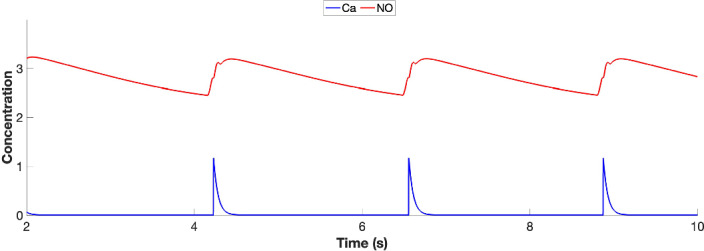
Concentration of NO and $\text {Ca}^{2+}$ in the WT collecting vessel at a point in the middle of the vessel wall over time

The concentrations of $\text {Ca}^{2+}$ and NO obtained for the WT collecting vessel are shown in Fig. 5, where we observe the expected regulatory behavior described in Padera et al. (2016). During each lymphatic contraction, increased flow activates endothelial NO synthase to produce NO. The elevated NO then attenuates the $\text {Ca}^{2+}$-dependent contraction, leading to vessel relaxation. As the vessel relaxes, shear stress and NO production decrease, allowing $\text {Ca}^{2+}$ levels to rise again and prepare the lymphangion for the next contraction cycle (Padera et al. 2016; Kunert et al. 2015).

**Fig. 6:**
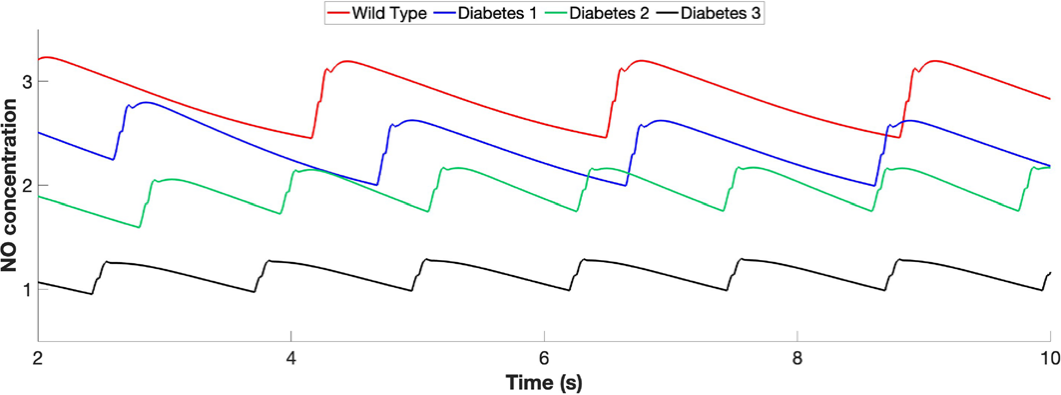
NO concentration at a point in the middle of the vessel wall over time

**Fig. 7:**
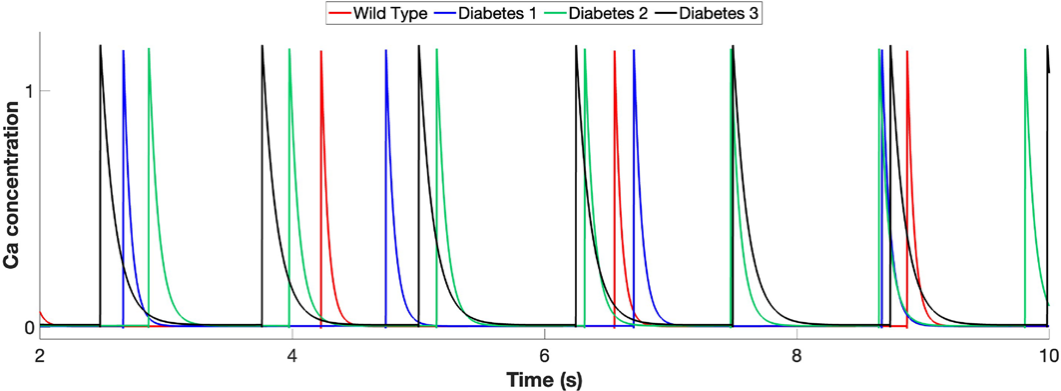
Concentration of $\text {Ca}^{2+}$ at a point in the middle of the vessel wall over time

A comparison of NO and $\text {Ca}^{2+}$ concentrations between the WT and diabetic cases is shown in Figs. 6 and 7, respectively. These concentrations are measured at a point in the middle of the vessel wall over time. As expected, the NO concentration decreases with the reduction in production rate. Interestingly, our results reveal that lower NO production rates lead to more frequent pulsations in both NO and $\text {Ca}^{2+}$ concentrations, while the peak $\text {Ca}^{2+}$ concentration remains relatively consistent across all cases.

**Fig. 8:**
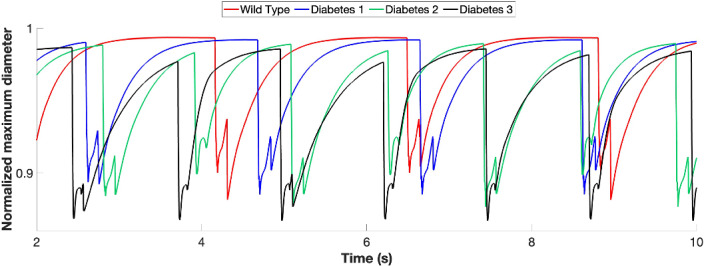
Normalized maximum diameter of the collecting lymphatic vessels over time

The $\text {Ca}^{2+}$ and NO concentrations, together with the fluid normal stress, govern the force that drives vessel wall contraction. Fig. 8 shows the maximum normalized diameter obtained for the WT and diabetic vessels over a period of 8 seconds.

**Fig. 9:**
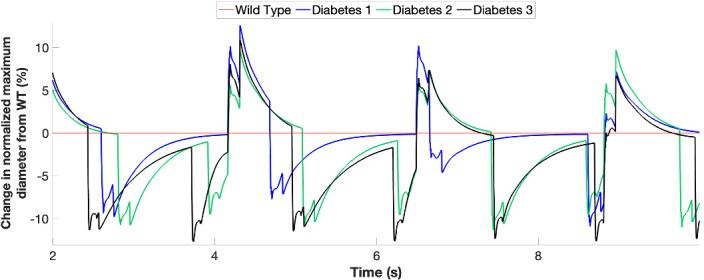
Change in normalized maximum diameter of diabetic collecting lymphatic vessels relative to WT (%)

Our results for the WT vessel are consistent with previous studies (Baish et al. 2016; Liao et al. 2011), which reported similar diameter variations during the contraction-relaxation cycle. In diabetic vessels, the reduced NO production rate leads to increased pumping frequency. Reducing the NO production rate by 25% and 50% increased contraction frequency by 17% and 99%, respectively. However, a further reduction to 75% yielded a smaller increase of 87.5%, suggesting that beyond a certain threshold, further NO depletion begins to suppress rather than enhance pumping frequency. The Diabetes 3 case, which has the lowest NO production rate, exhibits the largest amplitude changes in vessel diameter. This behavior is consistent with the expected role of NO as a vasodilator that promotes vessel relaxation. When NO levels are reduced, the attenuating effect on contraction is diminished, resulting in more pronounced diameter oscillations. Fig. 9 shows the change in normalized maximum diameter relative to WT, illustrating how the diabetic cases differ from the baseline in terms of vessel wall motion.

**Fig. 10:**
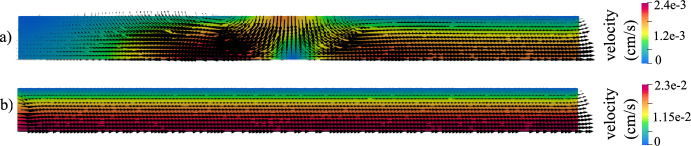
Visualization of the fluid velocity field during different phases. a) Contraction phase at $t = 4.22\,\text {s}$, with higher velocities observed in the central region. b) Relaxation phase at $t = 4.13\,\text {s}$, characterized by a unidirectional rightward flow

Figure 10 shows the fluid velocity field during contraction and relaxation phases, with color indicating velocity magnitude. During contraction, the vessel constricts at its midpoint, driving lymph fluid outward bidirectionally. During relaxation, as the vessel re-expands, the flow becomes unidirectional from left to right, consistent with the pressure gradient established across the lymphangion. These velocity patterns agree with observations reported in Li et al. (2019).

**Fig. 11:**
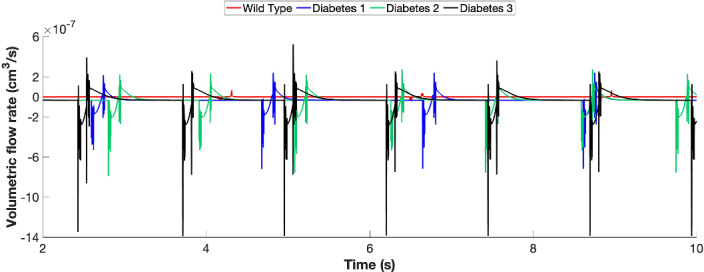
Volumetric flow rate of lymph fluid across the interface over time

We also compute the volumetric flow rate across the interface Γ to quantify the amount of fluid passing from the lymph vessel into the surrounding tissue. A comparison of the WT and diabetic collecting lymphatic vessels over an 8-second period is shown in Fig. 11. The negative values indicate fluid leaking out of the vessel into the tissue, which is particularly pronounced in the diabetic cases and consistent with increased fluid exchange associated with compromised lymphatic barrier function. All diabetic vessels exhibit flow rates increased by about two orders of magnitude compared to the WT case, reflecting substantially increased transvascular fluid exchange, driven by the higher hydraulic conductivity prescribed in the diabetic cases.

While Diabetes 1 and 2 cases show similar results, Diabetes 3 exhibits flow rate peaks 83% larger than the other diabetic cases. This enhanced leakage correlates with the lowest NO production rate in the Diabetes 3 case.

To quantify the net fluid exchange across the vessel wall, we compute the accumulated volume as the time integral of the normal flux over the 8-second interval. In both WT and diabetic cases, the accumulated volumes are negative (Table 4), indicating net fluid loss from the vessel lumen into the surrounding interstitium. Within the diabetic cases, the accumulated volume varies modestly. Diabetes 3 shows approximately 7% greater fluid loss than Diabetes 1, despite a 66.6% reduction in NO production, suggesting that hydraulic conductivity is the primary determinant of transvascular fluid exchange.

**Table 4:** The accumulated volume for different cases considered in this study

Parameter	Wild Type	Diabetes 1	Diabetes 2	Diabetes 3	Units
*V*	$-2.8143\cdot 10^{-9}$	$-2.7603\cdot 10^{-7}$	$-2.8124\cdot 10^{-7}$	$-2.9498\cdot 10^{-7}$	$\text {cm}^3$

These results align with experimental observations that collecting lymphatic vessels constitutively leak a portion of their transported fluid, and that compromised lymphatic barrier function under diabetic conditions leads to increased leakage into surrounding tissue (Scallan et al. 2015). Our model successfully reproduces this pathological behavior: the diabetic simulations predict significantly enhanced fluid exchange between the lymphatic vessel and interstitium compared to the WT condition.

### The effects of the Young’s modulus

To examine the effects of the vessel wall stiffness, we consider one of the diabetic cases, Diabetes 2, described in Table 2, where the Young’s modulus is equal $1\cdot 10^4 \, \text {dyn/} \text {cm}^2$. We then increase the Young’s modulus keeping all other parameters the same, resulting in two additional test cases labeled Diabetes $2^+$ and Diabetes $2^{++}$, with Young’s moduli of $1.5\cdot 10^4 \, \text {dyn/} \text {cm}^2$ and $2\cdot 10^4 \,\text {dyn/} \text {cm}^2$, respectively.

**Fig. 12:**
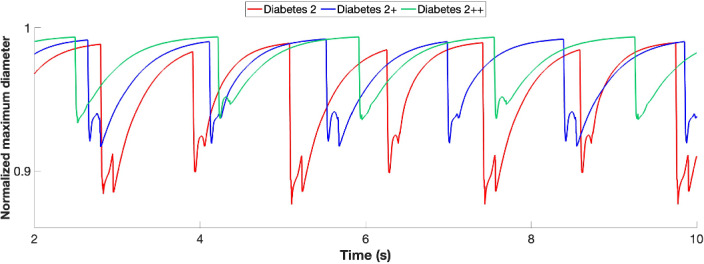
Normalized maximum diameter of the diabetic collecting lymphatic vessels

As expected, the increased stiffness leads to a decrease in the maximum normalized diameter, as shown in Fig. 12, and consistent with previous findings (Baish et al. 2016). Interestingly, we also observe that the pulsation frequency decreases with increasing Young’s modulus, with stiffer vessels exhibiting less frequent pulsations. Specifically, increasing Young’s modulus by 50% led to a 20% decrease in pulsation frequency and a 36% decrease in the amplitude, while doubling the Young’s modulus resulted in a 31% decrease in pulsation frequency and a 48% decrease in the amplitude.

**Fig. 13:**
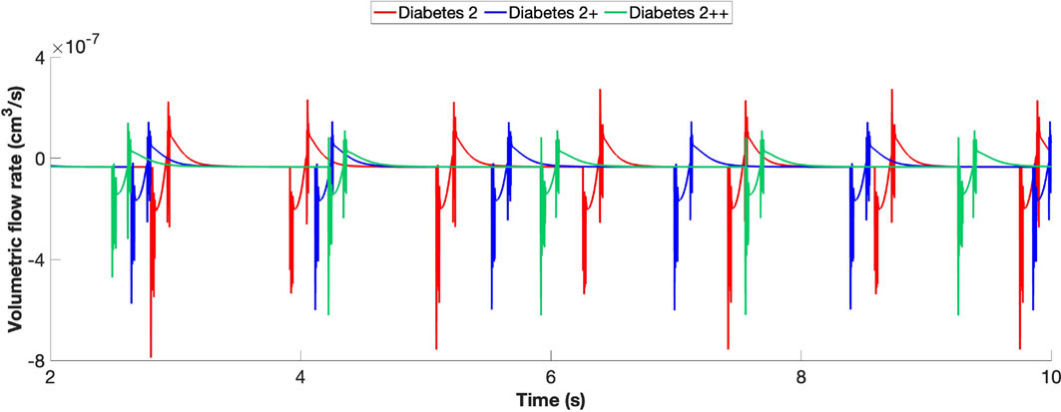
Volumetric flow rate of lymph fluid across the interface over time for the collecting lymphatic vessel affected by diabetes for different values of Young’s modulus..

We next examine how changes in Young’s modulus affect the volumetric flow rate across the vessel wall. The results for all three cases are presented in Fig. 13. During the contraction phase, we observe a slight reduction in flow rate as Young’s modulus increases. The accumulated volume over the 8-second interval is $-2.8124\cdot 10^{-7}\text {cm}^3$, $-2.8801\cdot 10^{-7}\text {cm}^3$, and $-2.7627\cdot 10^{-7}\text {cm}^3$ for Diabetes 2, Diabetes $2^+$, and Diabetes $2^{++}$, respectively. While these values show some variation, the differences are relatively small (approximately 2-3%), indicating that vessel stiffness has a modest effect on total fluid transport over this time interval. The primary effect of increased stiffness is thus a reduction in pulsation frequency rather than a substantial change in net fluid transport.

### The effects of the storage coefficient

To further assess the robustness of our findings, we perform a sensitivity analysis examining the effects of the storage coefficient. We again use the Diabetes 2 case from Table 2, where the storage coefficient is $c_0=10^{-5}$, and consider two additional values: $c_0=10^{-8}$ and $c_0=0$, which we refer to as Diabetes 2* and Diabetes 2**, respectively.

**Fig. 14:**
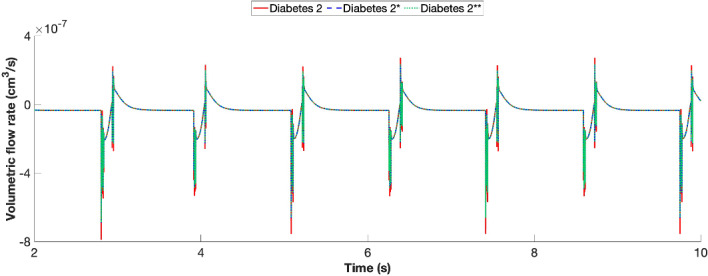
Volumetric flow rate of lymph fluid across the interface over time for the collecting lymphatic vessel affected by diabetes obtained using different values of the storage coefficient

We investigate how the storage coefficient influences the volumetric flow rate. Decreasing the storage coefficient results in a reduced volumetric flow rate, as shown in Fig. 14.

Notably, the flow rates for the two smallest values of the storage coefficient (cases Diabetes 2* and Diabetes 2**) are nearly identical, suggesting that the effect saturates at very low values.

**Fig. 15:**
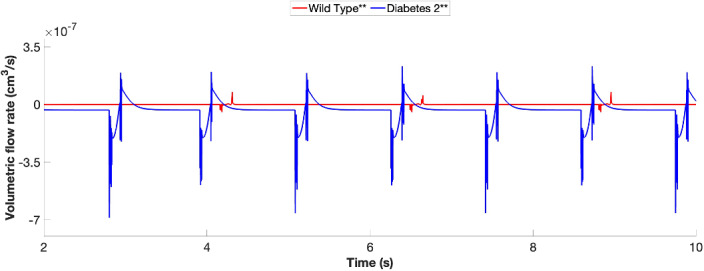
Volumetric flow rate of lymph fluid across the interface over time

To verify whether these changes affect our qualitative conclusions, we compare the Diabetes 2** case ($c_0=0$) to a modified WT case with $c_0=0$, which we refer to as WT**. The volumetric flow rates for these two cases are compared in Fig. 15.

The accumulated volume between 2 and 10 s is $-2.7936\cdot 10^{-9}\text {cm}^3$ for WT** and $-2.8121\cdot 10^{-7}\text {cm}^3$ for Diabetes 2**. These results demonstrate that, even when the storage coefficient is set to zero, fluid exchange across the vessel wall remains approximately two orders of magnitude smaller in the WT case than in the diabetic case. Comparing Fig.15 to Fig. 11, we observe that the relationship between the WT and diabetic cases is preserved. Furthermore, additional simulations indicate that the maximum normalized diameter is insensitive to the parameters varied here, and these results are therefore not shown. Thus, while small quantitative differences arise when varying $c_0$, the qualitative behavior remains consistent across all storage coefficient values examined.

### The effects of the hydraulic conductivity

To restore lymphatic function in diabetes, L-arginine levels were increased in Scallan et al. (2015), where L-arginine serves as the amino acid substrate for NO synthesis. The data in Scallan et al. (2015) suggested that restoring NO signaling in diabetes resolves lymphatic barrier dysfunction by lowering solute permeability. To investigate this hypothesis, we increase the NO production rate to match the WT value while maintaining the elevated permeability observed in the diabetic cases. This case is labeled Diabetes 4, with parameters summarized in Table 2.

**Fig. 16:**
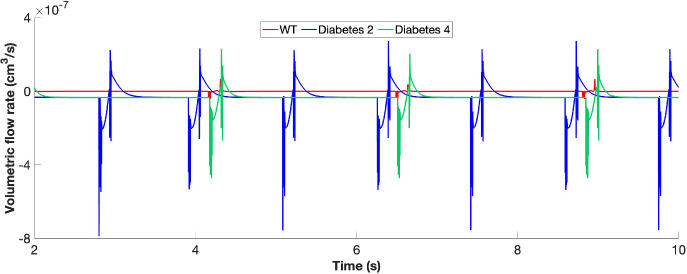
Volumetric lymph flowrate across the interface at the vessel wall over time

A comparison of the volumetric flow rates across the interface for the WT, Diabetes 2, and Diabetes 4 cases is shown in Fig. 16. As seen in the figure, the WT and Diabetes 4 cases exhibit the same pulsation frequency, consistent with the restored NO production rate in Diabetes 4. However, significantly increased fluid exchange across the vessel wall persists in both diabetic cases. Increasing the NO production to match that of the WT case still resulted in flowrates that are about two orders of magnitude larger than WT. The Diabetes 2 case, in which the NO production rate is additionally reduced, exhibits larger contraction amplitude and increased pulsation frequency compared to both the WT and Diabetes 4 cases. In particular, the contraction amplitude is about 60% larger in the Diabetes 2 case compared to Diabetes 4. This contrast suggests that hydraulic conductivity and NO production rate affect distinct aspects of lymphatic function and that restoring full lymphatic function in diabetic conditions requires the simultaneous normalization of both parameters, in agreement with the experimental observations of Scallan et al. (2015).

## Conclusion

We developed a mathematical model and numerical method to simulate lymph flow and fluid transfer between a collecting lymphatic vessel and the surrounding interstitium in both WT and diabetic cases. We used this framework to investigate how NO production rate, Young’s modulus, storage coefficient, and hydraulic conductivity affect lymphatic function. Our results reveal several key findings. First, we observe a disruption in the mechanobiological oscillator modulated by $\text {Ca}^{2+}$ and NO dynamics when the NO production rate becomes sufficiently low. The simulations demonstrate that diabetic conditions are associated with substantially increased fluid exchange across the collecting lymphatic vessel wall, with accumulated volumes approximately two orders of magnitude larger than the WT case. Second, the WT case exhibits higher pumping frequency compared to the diabetic cases, which is correlated with the decreased NO production rate. Third, increased Young’s modulus in the diabetic cases results in reduced vessel diameter and altered pulsation dynamics, with stiffer vessels exhibiting lower contraction frequency. Regarding the storage coefficient, for which experimental measurements are currently unavailable, we conducted a sensitivity analysis. While vessel diameter remains largely unaffected by changes in $c_0$, small differences in volumetric flow rate are observed. Importantly, our analysis confirms that the qualitative behavior and key conclusions remain consistent across all storage coefficient values examined, with quantitative differences being modest. Finally, our simulations indicate that therapeutic strategies targeting only NO production are insufficient to restore normal lymphatic function in diabetic conditions, as significant lymph leakage persists even when NO production is restored to WT levels. Instead, our results suggest that effective restoration of lymphatic function requires simultaneous normalization of both NO production rate and hydraulic conductivity to WT values. This finding has important implications for therapeutic interventions aimed at addressing lymphatic dysfunction in diabetes.

Several limitations of the present study merit discussion. First, the model is formulated in an idealized 2D geometry. Moreover, the present model does not explicitly represent the lymphatic valves, but instead implicitly captures their effect through the mechanobiological oscillator, as discussed in Sect. 2.1. An explicit representation of the valves would more accurately reproduce flow rectification and backflow suppression. However, this would give rise to a fluid-structure interaction problem with contact, which is challenging to approximate numerically. Extension to 3D geometries would require greater computational resources, but would enable a more realistic representation of the lymphangion. Second, while the model parameters are drawn from published experimental data, their calibration remains challenging. Parameters such as the NO decay rate or the storage coefficient are particularly difficult to measure directly in vivo, which motivated the sensitivity analysis presented in Sect. 3.3. Moving toward patient-specific simulations would additionally require medical imaging data for geometric reconstruction, combined with targeted measurements of metabolic markers relevant to diabetic lymphatic dysfunction, including NO bioavailability and interstitial fluid pressure. We regard these extensions as important avenues for future investigation.

## Data Availability

Data will be made available on reasonable request.

## Footnotes

1 Created in BioRender. Furkes, M. (2025) https://BioRender.com/hd6lcrj.

2 Created in BioRender. Furkes, M. (2025) https://BioRender.com/p80s5zf.
